# The Systemic Lupus Erythematosus Interventional Trials in Mainland China: A Continuous Challenge

**DOI:** 10.3389/fimmu.2022.848478

**Published:** 2022-04-11

**Authors:** Jingru Tian, Hang Zhou, Juan Liu, Feng Xiong, Ping Yi, Pengpeng Cao, Dorthy Fang, Bo Zhang, Qianjin Lu

**Affiliations:** ^1^ Institute of Dermatology, Chinese Academy of Medical Sciences and Peking Union Medical College, Nanjing, China; ^2^ Key Laboratory of Basic and Translational Research on Immune-Mediated Skin Diseases, Chinese Academy of Medical Sciences, Nanjing, China; ^3^ Jiangsu Key Laboratory of Molecular Biology for Skin Diseases and Sexually Transmitted Infections, Nanjing, China; ^4^ Department of Dermatology, Hunan Key Laboratory of Medical Epigenomics, The Second Xiangya Hospital, Central South University, Changsha, China; ^5^ Department of Molecular, Cellular, and Developmental Biology, Yale University, New Haven, CT, United States

**Keywords:** systemic lupus erythematosus, randomized clinical trials, cross-sectional study, review, China

## Abstract

**Objectives:**

More than a quarter of single-country systemic lupus erythematosus (SLE) interventional randomized clinical trials (RCTs) were conducted in China. To help develop management guidelines and set benchmarks for future SLE research, a systematic review of current trials is needed.

**Methods:**

We searched systematically three databases and four registries to summarize the interventional RCTs in mainland China and identify factors associated with participant loss. The internal validity of trials was assessed using the Cochrane risk-of-bias tool for assessing risk of bias. The odds ratio (OR) was defined as the ratio of the odds of less than 10% loss to follow-up in the presence or absence of different factors.

**Results:**

A total of 188 trials met our inclusion criteria, and 15·5% of trials conducted in mainland China ranked low risk of bias. Participant loss was significantly higher among trials that had a defined primary outcome or were registered {primary outcome identification (0·02 [0·00-0·23]) and registration (0·14 [0·03-0·69])}. Trials examining traditional Chinese medicine (TCM) pharmacological treatments had an 8·16-fold (8·16 [1·28-51·98]) higher probability of having low participant loss than trials examining non-TCM pharmacological treatment trials, and trials that did not report masking status had a 15·95-fold (15·95 [2·45-103·88]) higher probability of having low participant loss than open-label trials. In addition, published articles in Chinese also had higher probability of having low participant loss (5·39 [1·10-26·37]).

**Conclusion:**

SLE trials conducted in mainland China were of relatively poor quality. This situation, including nonrigorous design, lack of registration, and absence of compliance reporting, needs to be ameliorated. To maintain the fundamental repeatability and comparability of mainland China SLE RCTs, transparency of the clinical trial process and complete reporting of the trial data are crucial and urgently needed.

## Introduction

Despite the large patient population, very few large-scale epidemiological surveys have been conducted in mainland China to determine the changes in the disease signatures of SLE. Previous studies showed an estimated prevalence of 30–70/100,000, and male to female ratio of 1:10–12 (1–5). Although SLE in ethnic Chinese has distinct features in its clinical manifestations and outcomes compared with those of white populations of European ancestry (6), currently, Chinese guidelines for the management of SLE patients are similar to international guidelines partly due to the lack of evidence from high-quality RCTs involving ethnic Chinese patients that could provide evidence for clinical practice. Rigorously designed and effectively conducted RCTs are needed to face the continuing challenge of the rising SLE burden in tailoring specific treatment and prevention strategies in China (7–9), and a retrospective summary comparing Chinese SLE trials with worldwide studies can contribute to the development of Chinese lupus research.

The variability arising from participant loss has become a worldwide problem of profound magnitude and is one of the major factors contributing to therapeutic partial response or nonresponse (10–13). Our previous study found that the participant loss was distinctly influenced by national income of the trial-conducted country. In low and middle income countries including China, the participant loss between intervention and control groups was altered by trial registration, year of start, number of centers, number of participants, and primary outcome identification (14). Since more than a quarter of single-country SLE interventional RCTs were conducted in China and ranked relative low quality, whether follow these requirements may lead to highly heterogenized results of trials and disturb clinical decision (14). To comprehensively analyze clinical trials conducted in mainland China and to prevent unnecessary bias in the future, in this cross-sectional study, we systematically searched for published articles from English-language journals and for records of registered trials in clinical trial registries for SLE RCTs performed in China over the past three decades, including general information and specific trial characteristics to identify limitations in the conduct of the trials with the aim of improving SLE research.

## Materials and Methods

### Search Strategy

A systematic search of the scientific literature was performed for RCTs within published English-language articles from peer-reviewed journals and registered trials in clinical trials registries on May 4, 2021. This was carried out using a combination of keywords and search strategies with Medical Subject Headings (MeSH), three databases (PubMed, EMBASE, and Cochrane Library Central Register of Controlled Trials (CENTRAL)) and four registries (ClinicalTrials.gov of the US National Library of Medicine https://clinicaltrials.gov/ct2/home; International Standard Randomized Controlled Trial Number Register (ISRCTN) https://www.isrctn.com/; Australian and New Zealand Clinical Trials Registry (ANZCTR) http://www.anzctr.org.au/; and Chinese Clinical Trial Register (ChiCTR) http://www.chictr.org.cn/index.aspx) were included. Study selection as well as screening and data extraction methods are provided in **Supplementary Tables S1–6, Figures S1, 2**.

### Data Analysis

A qualitative synthesis of the types of interventions and sample characteristics of the included trials was performed. Risk of bias was assessed by version 2 of the Cochrane risk-of-bias tool for randomized trials (RoB 2). The OR was defined as the ratio of the odds of less than 10% loss to follow-up in the presence or absence of different factors. If the OR was greater than 1, the presence of the factor raised the odds of less than 10% loss to follow-up. Logistic regression was used to calculate crude and adjusted ORs, and subject (SLE or LN), intervention duration, and multinational factors were covariates. All ORs were calculated using SPSS version 25.

All statistical tests were two-sided. Ethics approval was not required for this study.

## Results

### Overview of SLE RCTs in Mainland China

**Figure 1:**
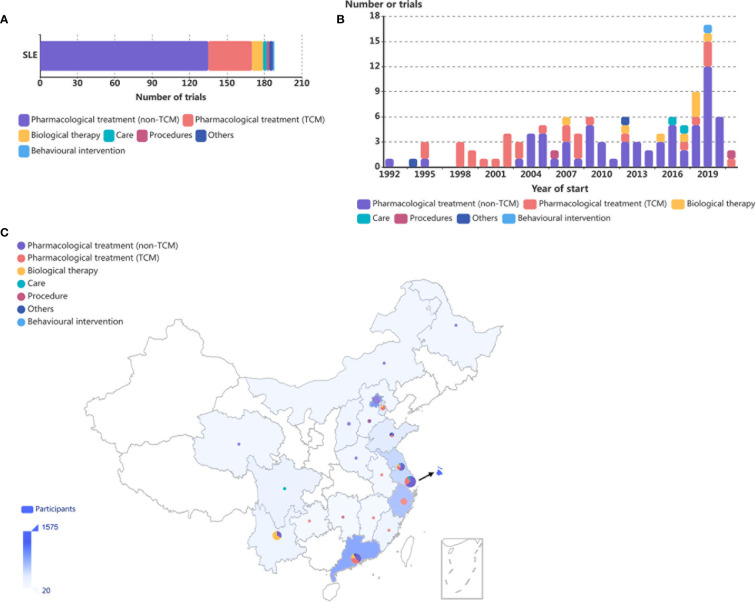
Distribution of SLE RCTs in mainland China. **(A)** Number of SLE RCTs conducted in mainland China in the last three decades by intervention categories. **(B)** Number of RCTs on SLE conducted in mainland China, by year of initiation and intervention categories. Eleven trials were not included because of lack of information on year of initiation. **(C)** Geographical distribution of the affiliations of primary investigators of RCTs by intervention categories and participants in SLE RCTs conducted in mainland China. The numbers of trials are summarized by province or municipality and are shown as pie charts, with larger charts indicating larger numbers of trials and the distribution of intervention categories represented within each geographical area. Numbers of participants are summarized by country and are represented by the shade of blue on the map, with deeper shades indicating larger numbers of participants.

A total of 188 RCTs that recruited participants in China were included in our study (**Supplementary Table S7**): 122 (64·9%) were performed within mainland China, 57 (30·3%) were multinational studies, and 96 (51·1%) were single-centre studies. Of the 122 multinational or single-country trials recruiting participants within mainland China, more than half (78, 63·9%) of the trials involved SLE patients without any other diagnosis (78, 62·4%); 31·1% (38) of the trials involved lupus nephritis (LN) patients, and 1·6% (2) of the trials involved patients who had neuropsychiatric lupus erythematosus (NPSLE). The type of intervention for the identified trials is summarized in **Figure 1A**, 86·9% (106) examined pharmacological treatments (TCM:33; non-TCM:73), followed by biological therapies (9, 7·4%), care (2, 1·6%), others (2, 1·6%), procedures (2, 1·6%), and behavioral interventions (1, 0·8%). The category “others” represents interventions that do not belong to any of the remaining categories; only 3 trials were enrolled in our study, and no further categorization could be made.

The numbers of RCTs involving patients with SLE conducted in mainland China showed an overall upward trend over the last three decades. Interestingly, among trials examining pharmacological treatments, before 2003, TCM was the main focus of the research, while the number of non-TCM trials increased rapidly and now constitutes the majority of all trials since 2004 (**Figure 1B**).

The geographical distribution of the 77 single-centre RCTs conducted in mainland China was uneven. Eastern China was most actively involved, accounting for 85·4% of all participants and 81·8% of all trials in SLE performed in mainland China (**Figure 1C**). Western, middle, and northeast China accounted for 8·4%, 5·3%, and 0·9% as well as 13·0%, 7·8%, and 1·3% of all participants and all trials, respectively (**Figure 1C**).

### Trial Design and Participant Characteristics of SLE RCTs in Mainland China

Since clinical trials on different intervention categories may have unique design considerations and characteristics, further descriptions based on intervention categories are necessary. However, among the 107 (56·9%) single-country trials performed within mainland China, 85% were pharmacological treatment trials; apart from pharmacological treatments and biological therapies, other intervention categories had only 1 or 2 trials each. The insufficient number of trials limits further intervention category-based analysis, and the following results are from all 107 trials, with the impact of intervention category mentioned when necessary.

**Figure 2:**
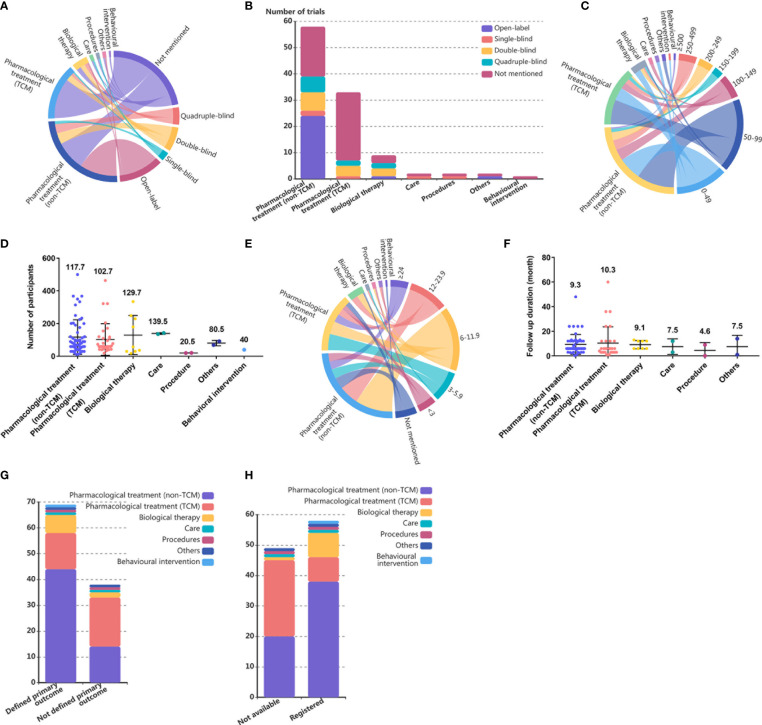
Study design of single country SLE RCTs in mainland China. **(A)** Chordal diagram of masking status of RCTs by intervention categories in SLE RCTs conducted in mainland China. The masking status and intervention categories each account for half of the circumference of the circle, with a longer arc length indicating more RCTs and a thicker line connecting the country and sample sizes indicating more RCTs. **(B)** Number of RCTs in SLE by intervention categories and masking status. **(C)** Chordal diagram of sample sizes of RCTs by intervention categories in SLE RCTs conducted in mainland China. The sample sizes and intervention categories each account for half of the circumference of the circle, with a longer arc length indicating more RCTs and a thicker line connecting the country and sample sizes indicating more RCTs. **(D)** Number of participants in SLE trials by intervention categories. Each dot represents one trial, and the value above dots represents the mean number of participants of one trial in each intervention category. **(E)** Chordal diagram of intervention duration in RCTs by intervention categories in SLE RCTs conducted in mainland China. The intervention duration and intervention categories each account for half of the circumference of the circle, with a longer arc length indicating more RCTs, and a thicker line connecting the country and sample sizes indicating more RCTs. **(F)** Number of RCTs in SLE by intervention categories and follow up duration. Each dot represents one trial, and the value above dots represents the mean follow-up duration of one trial in each intervention category. **(G)** Number of RCTs in SLE by intervention categories and primary outcome definition. **(H)** Number of RCTs in SLE by intervention categories and registration.

A total of 24·3% (26) of trials were open-label trials, 4·7% (5) were single-blinded, 13·1% (14) were double-blinded, and 9·3% (10) were quadruple-blinded, and 52 (48·6%) of the trials did not report masking status (**Figure 2A**). Compared to 67·2% of the non-TCM pharmacological treatment trials and 66·7% of the biological therapy trials, only 21·2% of the TCM pharmacological treatment trials reported masking status (**Figure 2B**).

Most of the trials conducted in mainland China had only small study populations: 16·8% (18) of the trials enrolled more than 200 people, and 65·4% (70) of the trials had fewer than 100 participants. Only trials examining care enrolled more than 100 participants (**Figures 2C, D**). The mean number of participants in trials examining pharmacological treatments was not significantly different from that in trials examining other intervention categories (**Figure 2D**).

A total of 58·9% (63) of the trials had a duration of less than 12 months. The intervention duration of RCTs was more distributed at 3 months (15, 14·0%), 6 months (32, 29·9%), and 12 months (16, 15·0%), and no significant difference in mean follow-up duration was found among intervention categories (**Figures 2E, F**). A total of 11·2% (12) of the trials did not mention the follow-up duration, and the proportion of trials with unknown follow-up duration was similar among trials examining non-TCM pharmacological treatment (12·1%), TCM pharmacological treatment (9·1%), and biological therapies (11·1%) (**Figure 2E**).

A total of 35·5% (38) of the trials did not define the primary outcome, and among all intervention categories, TCM pharmacological treatments (42·4%) had the lowest rate of primary outcome definition (**Figure 2G**).

A total of 54·2% (58) of the published single-country RCTs conducted in mainland China reported trial registration identification numbers, 88·9% of biological therapy trials had registration records, followed by trials studying non-TCM pharmacological treatments, while the rate of registration for trials examining TCM pharmacological treatments was only 24·2% (**Figure 2H**).

Among the multinational studies, all 57 trials examined non-TCM pharmacological treatments, and 70·2% (40) had over 200 participants. Double-blind study designs (31, 54·4%) was the most frequently used among all masking status types, followed by quadruple-blind (17, 29·8%), open-label (6, 10·5%), and triple-blind (3, 5·3%) designs. The intervention duration ranged from 2·4 to 52 months, and 22·8% (13) of the trials ran for 13 months. All trials identified the primary outcome. Overall, multinational trials showed a more rigorous study design than that found in single country trials.

### Outcome Reporting and Withdrawal Rates of SLE RCTs in Mainland China

**Figure 3:**
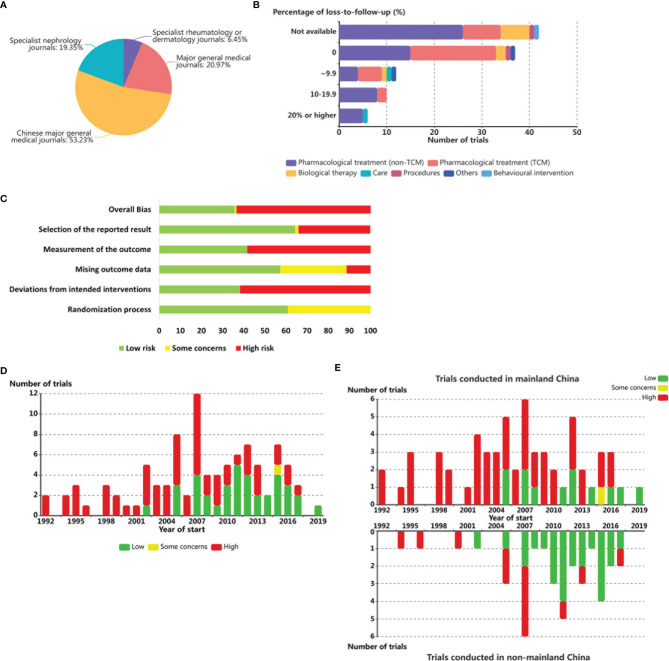
Intervention duration and percentage of loss-to-follow-up among LE RCTs worldwide. **(A)** Number of published SLE RCTs by journal categories. **(B)** Number of SLE RCT by intervention categories and percentage of participant loss. **(C)** Risk of bias summary (Low, some concerns, and high). Each risk of bias item is shown as percentages across all included studies. **(D)** Number of SLE RCTs by year of start and overall risk of bias. **(E)** Number of SLE RCTs conducted in mainland China (above) or non-mainland China (below) by year of start and overall risk of bias.

In terms of outcome reporting, for registered clinical trials, compliance reporting was defined as submitting results within 1 year of completion and only 27·3% (3 of 11) of mainland China’s RCTs conducted during the past three decades and listed on ClinicalTrials.gov met this requirement. Among 62 published trials, 53·2% were published in Chinese journals. Of these, 6·5% were published in specialized rheumatology or dermatology journals rather than in major general medical journals (74·2%), and the remaining 19·4% of the trials were published in specialized nephrology journals (**Figure 3A**).

Among the 65 (60·7%) trials conducted in mainland China with available data, loss to follow-up was 0% in 56·9% (37) of the trials, more than 0% and less than 10% in 18·5% (12) of the trials, 10·0%-19·9% in 15.4% (10) of the trials, and 20% or higher in 9·2% (6) of the trials. Multinational studies reported higher overall percentages of participant loss, with 0% in 0 trial, more than 0% and less than 10% in 15·2% (5) of the trials, 10·0%-19·9% in 39·4% (13) of the trials, and >20% in 45·5% (15) of the trials (**Figure 3B**).

### Factors Associated With Withdrawal Rates From SLE RCTs

A total of 115 trials conducted in China with available results were involved in the analyses. Risk of bias assessments are shown in **Figure 3C** and **Supplementary Figure S3**. The overall bias of RCTs among patients with SLE conducted in China showed an overall declining trend during the last three decades (**Figure 3D**); however, this mitigating trend was mainly brought about by non-mainland Chinese trials (**Figure 3E**).

Analyses were performed to determine the factors associated with loss of participants prior to the end of the individual studies. We defined a low percentage of loss to follow-up as less than 10% of the participants failing to complete the study; crude and adjusted multivariate ORs across all explanatory variables are presented in **Table 1**. In the adjusted analyses, loss to follow-up was significantly higher in trials that had a defined primary outcome or that were registered (primary outcome identification: OR 0·02 [0·02-0·23] and registration: OR 0·14 [0·03-0·69]). Trials examining TCM pharmacological treatments had an 8.16-fold (OR 8·16 [1·28-51·98]) higher probability of having low rates of loss to follow-up compared to trials examining non-TCM pharmacological treatment, and trials that did not report masking status had a 15·95-fold (OR 15·95 [2·45-103·88]) higher probability of having low rates of loss to follow-up compared to open-label trials. In addition, published articles in Chinese also had a 5·39-fold higher probability of having low rates of loss to follow-up compared to articles published in English language journals (OR 5·39 [1·10-26·37]).

**Table 1:** Crude and adjusted ORs for factors associated with less than 10% participant loss in single-country studies conducted in mainland China.

Categories	Loss-to-follow-up crude OR (95% CI; p value)	Loss-to-follow-up adjusted OR (95% CI; p value)
**Data source**		
** Published articles**	1 (ref)	1 (ref)
** Published articles in Chinese**	7·06 (1·74-28·57; 0·006)	5·39 (1·10-26·37; 0·04)
** Clinical trials registries**	1·41 (0·11-17·40; 0·79)	–
**Compliance reporting**		
** No**	1 (ref)	1 (ref)
** Yes**	4·00 (0·21-75·66; 0·36)	-
**Trial registration**		
** No**	1 (ref)	1 (ref)
** Yes**	0·13 (0·04-0·46; 0·002)	0·14 (0·03-0·69; 0·02)
**Center**		
** Single center**	1 (ref)	1 (ref)
** Multiple centers**	0·25 (0·06-1·02; 0·05)	0·46 (0·09-2·29; 0·34)
**Year of start**		
** 1995-2001**	2·25 (0·41-12·44; 0·35)	1·69 (0·22-13·19; 0·62)
** 2002-2011**	1 (ref)	1 (ref)
** 2012-2021**	0·72 (0·18-2·82; 0·64)	0·23 (0·03-1·65; 0·14)
** Not available**	10·66 (0·57-200·42; 0·11)	–
**No. of participants**		
** <50**	1 (ref)	1 (ref)
** 50-99**	1·49 (0·34-6·44; 0·60)	1·02 (0·12-8·67; 0·99)
** 100-199**	0·36 (0·06-2·00; 0·24)	1.62 (0·23-11·18; 0·63)
** ≥200**	0·38 (0·06-2·46; 0·31)	0·45 (0·05-4·35; 0·49)
**Subjects**		
** SLE**	1 (ref)	1 (ref)
** LN**	0.20 (0.06-10.67; 0.009)	0.37 (0.09-1.57; 0.18)
**Blinding**		
** Open label**	1 (ref)	1 (ref)
** Single blind**	11·31 (0·50-256·20; 0·13)	-
** Double blind**	10·00 (0·96-104·49; 0·05)	9·35 (0·68-129·47; 0·10)
** Not mentioned**	11·33 (2·85-45·07; 0·0006)	15·95 (2·45-103·88; 0·004)
**Intervention**		
** Non-TCM pharmacological treatment**	1 (ref)	1 (ref)
** TCM pharmacological treatment**	7·87 (1·58-39·28; 0·01)	8·16 (1·28-51·98; 0·03)
** Biological therapy**	4·85 (0·23-101·65; 0·31)	-
**Intervention duration (months)**		
** <5.9**	28·85 (1·55-537·96; 0·02)	-
** 6-11.9**	1 (ref)	1 (ref)
** 12-23.9**	1·25 (0·31-4·98; 0·76)	0·81 (0·17-3·86; 0·79)
** ≥24**	1·73 (0·27-10·97; 0·56)	1·06 (0·14-7·95; 0·96)
**Primary outcome identification**		
** No**	1 (ref)	1 (ref)
** Yes**	0·09 (0·02-0·37; 0·0009)	0·02 (0·00-0·23; 0·002)
**Overall risk of bias**		
** Low risk of bias**	1·24 (0·13-12·07; 0·85)	1·48 (0·11-3·16; 0·77)
** High risk of bias**	1 (ref)	1 (ref)

To compare the single-country study conducted in mainland China with other studies involving Chinese participants, the same method was used to calculate crude and adjusted multivariate ORs (**Table 2**). In the adjusted analyses, loss to follow-up was significantly higher only in trials conducted in multiple countries (OR 0·04 [0·01-0·23]).

**Table 2:** Crude and adjusted ORs for factors associated with less than 10% participant loss in multinational studies or in single-country studies conducted in non-mainland China.

Categories	Loss-to-follow-up crude OR (95% CI; p value)	Loss-to-follow-up adjusted OR (95% CI; p value)
**Data source**		
** Published articles**	1 (ref)	1 (ref)
** Published articles in Chinese**	-	-
** Clinical trials registries**	0·18 (0·02-1·55; 0·12)	0·44 (0·04-4·87; 0·48)
**Compliance reporting**		
** No**	1 (ref)	1 (ref)
** Yes**	0·40 (0·04-3·84; 0·42)	1·21 (0·07-22·55; 0·90)
**Trial registration**		
** No**	1 (ref)	1 (ref)
** Yes**	0·10 (0·02-0·47; 0·003)	0·60 (0·06-5·71; 0·66)
**Multinational**		
** No**	1 (ref)	1 (ref)
** Yes**	0·05 (0·01-0·24; 0·0001)	0·04 (0·01-0·23; 0·0004)
**Center**		
** Single center**	1 (ref)	1 (ref)
** Multiple centers**	0·04 (0·01-0·24; 0·0003)	0·09 (0·01-1·45; 0·09)
**Year of start**		
** 1995-2001**	2·83 (0·15-52·74; 0·49)	1·68 (0·02-145·76; 0·82)
** 2002-2011**	1 (ref)	1 (ref)
** 2012-2021**	0·61 (0·13-2·88; 0·53)	1·08 (0·11-10·91; 0·95)
**Not available**	45·77 (2·30-910·95; 0·01)	-
**No. of participants**		
** <50**	1 (ref)	1 (ref)
** 50-99**	0·50 (0·08-3·08; 0·46)	1·12 (0·10-12·90; 0·93)
** 100-199**	0·50 (0·05-4·58; 0·54)	0·46 (0·03-6·32; 0·56)
** ≥200**	0·01 (0·00-0·17; 0·002)	–
**Subjects**		
** SLE**	1 (ref)	1 (ref)
** LN**	1·44 (0·40-5·11; 0·57)	1·13 (0·22-5·77; 0·88)
**Blinding**		
** Open label**	1 (ref)	1 (ref)
** Single blind**	0·09 (0·00-2·51; 0·16)	–
** Double blind**	0·17 (0·04-0·83; 0·03)	0·05 (0·00-1·76; 0·10)
** Quadruple blind**	0·06 (0·01-0·74; 0·03)	0·02 (0·00-1·80; 0·09)
** Not mentioned**	2·33 (0·09-62·68; 0·61)	-
**Intervention**		
** Non-TCM pharmacological treatment**	1 (ref)	1 (ref)
** TCM pharmacological treatment**	1·76 (0·10-30·05; 0·69)	–
**Intervention duration (months)**		
** <5.9**	0·75 (0·10-5·69; 0·78)	0·36 (0·02-6·17; 0·48)
** 6-11.9**	1 (ref)	1 (ref)
** 12-23.9**	0·30 (0·07-1·29; 0·11)	0·15 (0·02-1·09; 0·06)
** ≥24**	0·90 (0·18-4·56; 0·90)	0·23 (0·02-2·36; 0·21)
**Primary outcome identification**		
** No**	1 (ref)	1 (ref)
** Yes**	0·06 (0·01-0·59; 0·02)	0·34 (0·02-5·61; 0·45)
**Overall risk of bias**		
** Low risk of bias**	0·19 (0·05-0·70; 0·01)	0·30 (0·03-2·77; 0·29)
** High risk of bias**	1 (ref)`	1 (ref)

## Discussion

Over the past 30 years, a total of 188 interventional SLE RCTs conducted in China have been reported. However, the quality of these RCTs and their reporting is of great concern. Most of the trials were missing one or more important characteristics, such as primary outcome definition, masking status, names of the involved institutions, or intervention duration; this was especially the case for the 122 trials conducted in mainland China. RCTs are commonly regarded as the highest level of evidence to support clinical decisions, but the relatively high risk of bias among the trials conducted in mainland China means caution must be used in interpreting the results, since biases directly impact the validity of the findings, and may lead to an exaggeration of treatment effects (15–17).

Although pharmaceutical interventions dominate lupus control measures, programs using provider strategies or education or reminders for patients can further improve lupus disease control and patient quality of life (18–22). However, interventional RCTs in mainland China focus on pharmacologic and biologic treatments, while the absence of interventions involving self-management, diet, education, exercise, and care reflects ignorance of the importance of comprehensive management for SLE patients among physicians in mainland China. Even in clinical practice, the importance of education and self-management of patients has been largely underestimated in mainland China (23–25), and it is necessary for researchers to explore localized nonpharmaceutical interventions in terms of nursing, education, and patient self-management. Based on that, coordinated efforts of physicians to establish a comprehensive and efficient clinical patient management system would support medication adherence, save medical resources, and help patients control their diseases.

During a trial, participant withdrawal almost always happens to some extent, and a careful interpretation of participant loss is required, since different rates of loss to follow-up, or losses of different types of participants, may change the characteristics of the groups, irrespective of the exposure or intervention (26). However, an abnormally low loss to follow-up is also worthy of vigilance; 52·1% of the trials in mainland China reported no participant loss during the trial, while the number was only 11·4% among trials in non-mainland China. Besides, China and the United States led in numbers of single-country interventional SLE RCTs worldwide, however, trials conducted in the United States had about 2.53-fold higher rates of participant loss per month compared to China (14). This extremely low participant loss rate may reflect potential bias, such as loose exclusion criteria, incomplete trial records, inaccurate result statistics, publication bias, or concealment of results reporting. Prospective registration of clinical trials is necessary to generate more transparent research, and compliance reporting imposes certain normative constraints on the trial results; together they sustain the validity of evidence-based practice and assure the availability of reliable data (27). In our study, among trials conducted in mainland China, registered studies and those that reported compliance had a significantly higher possibility of participant loss, with a 7- and 50-fold differences, respectively. However, these differences did not hold for non-mainland China trials, indicating that the impact of registration and compliance reporting is unique and of great importance for mainland China’s RCTs. More detailed subgroup analysis is limited by the number of trials. However, combined with the risk of bias assessment, the majority of SLE trials in mainland China lacked mandatory prospective trial design and strict execution, which resulted in potential internal heterogeneity and may eventually confound the universal clinical usefulness of the data generated.

In summary, the deficiencies of SLE RCTs in mainland China are mainly reflected in two aspects: the narrow intervention category and poor trial quality. Our suggestions for future mainland China trials are listed in **Table 3**.

**Table 3:** Requirements to improve SLE trial quality in mainland China.

Requirements
**1. Increased RCTs of nonpharmacological intervention categories**
**2. Prospective registration**
**3. Compliance reporting on registries**
**4. Improve trial design** ** Major objective** ** Sample estimate** ** Randomization method** ** Masking status** ** Inclusion and exclusion criteria** ** Primary outcome** ** Statistical methods** ** Intervention duration or termination sign** ** Location(s)**
**5. Detailed result record**
**6. Standardize the publication form of trials to prevent omission of information**

The number of SLE RCTs in mainland China was at the forefront of the world; however, despite this surge in the number of trials, the loose design, unbalanced intervention categories, and poor trial quality are concerning. By bringing awareness to the noncompliance with trial registration and reporting results that shaken the cornerstone of SLE RCTs in mainland China, our systematic review has important implications for future lupus research in mainland China. To generate high-quality evidence that can contribute to clinical practice, we must promote the standardization of clinical trial design and results reporting.

## Data Availability Statement

The original contributions presented in the study are included in the article/**Supplementary Material**. Further inquiries can be directed to the corresponding author.

## Author Contributions

QL and JT conceived of the study. JT and HZ developed the protocol. JT and HZ did the literature search. JT, PC, PY, FX, HZ, BZ and JL appraised study quality, and extracted and analyzed the data. JT, DF, and QL interpreted the data. JT wrote the first draft of the article. QL revised the draft paper. All authors contributed to the article and approved the submitted version.

## Funding

We acknowledge for the Non-profit Central Research Institute Fund of Chinese Academy of Medical Sciences (No. 2020-RC320-003), National Natural Science Foundation of China (No. 81830097).

## Conflict of Interest

The authors declare that the research was conducted in the absence of any commercial or financial relationships that could be construed as a potential conflict of interest.

## Publisher’s Note

All claims expressed in this article are solely those of the authors and do not necessarily represent those of their affiliated organizations, or those of the publisher, the editors and the reviewers. Any product that may be evaluated in this article, or claim that may be made by its manufacturer, is not guaranteed or endorsed by the publisher.
