# Supplementary material for: The Systemic Lupus Erythematosus Interventional Trials in Mainland China: A Continuous Challenge
Source: Front Immunol. 2022 Apr 11;13:848478. doi: 10.3389/fimmu.2022.848478 (PMC9035534; doi:10.3389/fimmu.2022.848478)
Supplement: Supplementary file 1 [file DataSheet_1.pdf]

SUPPLEMENTARY MATERIAL

The systemic lupus erythematosus interventional trials in mainland China: a continuous challenge

Jingru Tian, M.D.[1,2,3], Hang Zhou, M.D.[4], Juan Liu, M.D.[4], Feng Xiong, M.D.[4], Ping Yi, M.D.[4], Pengpeng Cao, M.D.[4], Dorthy Fang, B.S.[5], Bo Zhang, M.D.[1], and Qianjin Lu, M.D.[1,2,3*]

[1]Institute of Dermatology, Chinese Academy of Medical Sciences and Peking Union Medical College, Nanjing, China

[2]Key Laboratory of Basic and Translational Research on Immune-Mediated Skin Diseases, Chinese Academy of Medical Sciences, Nanjing, China

[3]Jiangsu Key Laboratory of Molecular Biology for Skin Diseases and Sexually Transmitted Infections, Nanjing, China

[4]Department of Dermatology, Hunan Key Laboratory of Medical Epigenomics, the Second Xiangya Hospital, Central South University, Changsha, Hunan, China

[5]Department of Molecular, Cellular, and Developmental Biology, Yale University, New Haven, CT, USA

* Correspondence should be addressed to:
Qianjin Lu, 12 Jiangwangmiao Street, Xuanwu, Nanjing, Jiangsu, China 210042.
Telephone: 025-85478999; Fax: 025-85414477;
Email: qianlu5860@pumcderm.cams.cn
ORCiD iD: 0000-0002-4192-4897

Appendices index

Appendix 1 Objectives and specific aims....3

Appendix 2 Detailed methods....4

Appendix 3 Search strategy....9

Appendix 4 Literature search and selection....12

Appendix 5 Data abstraction form....14

Appendix 6. Characteristics of included RCTs....15

Appendix 7. Risk of bias assessments....17

Appendix 1 Objectives and specific aims

The objective of the proposed study is to provide an overview of randomized clinical trials (RCTs) of Systemic lupus erythematosus (SLE) in China by systematically reviewing all published or registered RCTs of LE during the past three decades.

Aim 1: to provide overall information of LE RCTs in mainland China such as numbers and percentages of RCTs using different interventions, numbers of RCTs started in each year, and the geographical distribution of primary investigator, et al. and to describe specific trial characteristics such as sample size, masking status, intervention duration, loss to follow-up, et al.

Aim 2: to determine various affecting participant loss to uncover the potential bias of SLE RCTs conducted in mainland China.

Aim 3: to identify limitations in SLE RCTs conducted in mainland China to inform future research and improve trial quality.

Significance and Background

China has huge number of SLE RCTs, while Chinese guidelines for the management of SLE patients are similar to international guidelines partly due to the lack of evidence from high-quality RCTs involving ethnic Chinese patients that could provide evidence for clinical practice. To comprehensively analyze clinical trials conducted in mainland China and to prevent unnecessary bias in the future, we systematically searched for published articles and for records of registered trials in clinical trial registries for SLE RCTs performed in China over the past three decades.

Appendix 2 Detailed methods

a. Search strategy

Two investigators (JR.T and H.Z) searched published articles and clinical trial registry records, appraised studies on eligibility, and extracted data independently. Discrepancies were discussed and agreed by consensus.

The search for RCTs included published articles from peer-reviewed English-language journals and registered trials in clinical trials registries, both up to May 4, 2021 and without start date restriction. The published articles were searched in literature databases including the PubMed, EMBASE, and Cochrane Library Central Register of Controlled Trials (CENTRAL). The MeSH and keyword search terms associated with lupus were used in each database. In order not to miss out on potentially useful articles, references cited in relevant reviews were also searched manually. RCTs published in Chinese medical journals were also included.

Records of registered RCTs were collected from 4 publicly available web-based clinical trials registries, including the ClinicalTrials.gov of the US National Library of Medicine, the International Standard Randomised Controlled Trial Number Register (ISRCTN), the Australian and New Zealand Clinical Trials Registry (ANZCTR), and the Chinese Clinical Trial Register. The keyword search term “lupus” was entered combined with other specific filtering options in advanced search function for ‘Country’, ‘Study type’, and ‘Current status’ et al. in searching for eligible RCTs.

b. Study selection

We evaluated published articles at the title or abstract level, with divergences resolved after consensus by two independent investigators. If potentially relevant, we evaluated them as complete reports according to prespecified selection criteria. For both published articles and registered records, trials were included if they enrolled subjects with lupus patients, and randomly assigned patients to different intervention groups. We excluded studies which are: 1) non-human studies; 2) observational studies; 3) studies without randomization or intervention groups; 4) studies not conducted in patients with lupus; 5) studies without ethics committee approval. In addition, published articles which are: 1) not in the English language or not in Chinese full text; 2) without full text (i.e. abstracts and conference proceedings) or not reporting original studies (i.e. narrative reviews, meta-analyses, editorials, commentaries, protocols, guidelines, or perspectives); or 3) duplicate reports and registration were also excluded. The search

process of literature from published articles and records from clinical trial registries are shown below.

c. Data extraction

Two investigators independently extracted information on characteristics of each included studies, including general information (author, publication year, registration ID, year of start, domestic or multinational, single- or multi-center, affiliations of primary investigators), participant characteristics (subject type, number of participants, loss to follow-up), study intervention (measures of intervention or control, duration, blinding), and primary outcomes. Some information of participant characteristics was not available for multinational trials because they did not provide information separately for participants in individual countries. Extracted data from published articles and records from clinical trials registries were entered separately into two piloted spreadsheets, and then combined together matched by the registration ID or other information if the registration ID was unavailable. For studies with data available from both sources, data from published articles were used. Potential duplicate registry entries were searched for by matching on important trial characteristics including year of start, affiliation of primary investigator, subject category, number of participants, interventions, and primary outcome. Published trials which did not include a trial registration ID was considered not registered.

The following information will be extracted from each included trial.

1 General information

- **1.1 Data source:** 'clinical trial registry', 'published articles', or 'published articles in Chinese'.
- **1.2 Author, year of publication:** the first author and publication year of the trial from published articles. For trials in the registries, name of the registers including 'ClinicalTrials.gov', 'International Standard Randomised Controlled Trial Number Register', Australian New Zealand Clinical Trials Registry', and 'Chinese Clinical Trial Registry' will be used.
- **1.3 Registration ID:** the registered number of the trial. For trials without registration ID, 'not available' will be used.

2 Trial information

- **2.1 Year of start:** the start year of the trial if it is available, otherwise 'not mentioned' will be used.

2.2 **Multinational study:** 'Yes' if the trial is a multinational study, or 'No' if the trial was conducted entirely in one country.

2.3 **Affiliation of primary investigator:** The affiliation of the primary investigator can be found in registries. For published articles, the affiliation of the corresponding author will be used. The last corresponding author will be chosen if there are multiple corresponding authors.

2.4 **Single or Multicenter:** 'Single center' if it is a single-center study, 'Multicenter' if the trial is conducted at ≥2 centers, or 'Not mentioned' if it is not recorded.

2.5 **Primary outcome:** the primary outcome identified in the included trial. For trials which list several outcomes without identification of the primary outcome, all the reported outcomes will be extracted and 'primary outcome not identified' will be noted.

3 **Participant characteristics**

3.1 **Subjects:** 'SLE' if subjects are patients with systemic lupus erythematosus; 'JSLE' if subjects are patients with juvenile-onset systemic lupus erythematosus; 'SCLE' if subjects are patients with subacute cutaneous lupus erythematosus; 'LN' if subjects are patients with lupus nephritis; 'MLN' if subjects are patients with membranous lupus nephritis; 'DPSLE' if subjects are patients with diffuse proliferative lupus nephritis; 'NPSLE' if subjects are patients with neuropsychiatric Lupus Erythematosus.

3.2 **Number of participants:** the number of randomized subjects in published articles, or the number of estimated enrollments for ongoing trials and the number of actual enrollments for completed trials in the registries.

3.3 **Number of participants loss to follow-up:** the number of participants who did not complete the follow-up. 'not available' will be used for registered ongoing trials.

3.4 **Percentage of loss-to-follow-up (%):** calculated by 'Number of participants loss to follow-up' divided by 'Number of participants'. 'not available' will be used for registered ongoing trials.

3.5 **Age duration (years):** the age duration in years of participants. 'Not mentioned' if it is not recorded or only has average age.

3.6 **Country and area:** the country and area where the clinical trial is located. For trials which are multinational studies, all the reported locations will be extracted.

4 **Study intervention**

4.1 **Intervention categories:** including 'Pharmacological treatment', 'Behavioral intervention', 'Dietary supplement', 'Biological therapy', 'Device', 'Procedure', and 'Others'.

4.2 **Intervention:** interventions (control) in the included trial.

4.3 **Intervention duration (months):** the intervention duration in months for completed trials. 'not available' will be used for ongoing registered trials or if information is not provided.

4.4 **Blinding:** including 'Single-blind', 'Double-blind', 'Open-label', or other types of blinding (triple-blind or quadruple-blind) if it is available. 'Not mentioned' if information on blinding is not provided.

5 **Reference:** the reference for published articles and URL for registered trials.

d. Study categorization

We included RCTs conducted in subjects with SLE. We further classified included RCTs according to interventions. We referred classifications adapted from the ClinicalTrials.gov registry, which has 7 categories of intervention including pharmacological treatment, behavioral intervention, dietary supplement, biological therapy, procedure, device, and others. We also adopted care as an independent intervention category. The following table 2 listed the detail information.

Table S1. The classification of intervention categories

Categories	Interventions
**Pharmacological treatment**	
Chemical drugs and biologicals	This includes trials which evaluate the effects of monotherapy of glucocorticoids, immunosuppressants, antimalarial drugs, biologicals, combination therapy of glucocorticoids and/or antimalarial drugs and other immunosuppressants and/or biologicals, and other chemical drugs such as docosahexaenoic acid, etc.
Traditional Chinese medicine	This includes trials which evaluate the effects of herbal compound formula and herbal concentrate-granules.
Antibodies	This includes trials which evaluate the effects of humanized monoclonal antibody against different targets.

Vaccines	This includes trials which evaluate the effects or safety of vaccines, such as herpes zoster vaccine, etc.
**Behavioral intervention**	
Diet intervention	Intervention using diet such as the low glycemic index diet, the low carbohydrate diet, the calorie restriction diet, etc
Exercise intervention	Intervention using exercise such as the aerobic and resistance exercise, high-intensity interval exercise, motion/muscle strengthening, etc.
Education	Intervention using group-based or individualized health educational program for LE management such as drug usage, telephone counseling intervention, cognitive behavioral intervention, lifestyle modification, etc.
**Dietary supplement**	Intervention using dietary supplement such as health products, fish oil, vitamins, etc.
**Biological therapy**	Intervention using stem cell therapy.
**Device**	Intervention using high-intensity laser device, needle-free injectors, autoinjector, foot orthoses, intrauterine device, etc.
**Procedure**	Intervention using plasmapheresis, acupuncture, laser coagulation, laser photocoagulation, surgery, etc.
**Care**	Intervention using different kind of care.
**Others**	Interventions not belong to any of the above categories are categorized as‘others’ such as motivational interviewing, sleep intervention, digital therapeutic intervention, mobile/web-based intervention, genetic, sunscreens , etc.

Appendix 3 Search strategies

Table S2. The search strategy in PubMed (Medline)

#	Terms	Quotes
**4**	#1 AND #2 AND #3	**2.367**
**3**	(randomized controlled trial [pt] OR controlled clinical trial [pt] OR randomized [tiab] OR placebo [tiab] OR clinical trials as topic [mesh: noexp] OR randomly [tiab] OR trial [ti]) NOT (animals [mh] NOT humans [mh])	**1.343.564**
**2**	("Therapeutics" [Mesh] OR "therapy" [Subheading] OR "prevention and control" [Subheading] OR Intervention OR prevention)	**15.006.631**
**1**	((((((("Lupus Erythematosus, Systemic"[Mesh] OR Systemic Lupus Erythematosus OR Lupus Erythematosus Disseminatus OR Libman-Sacks Disease OR Disease, Libman-Sacks OR Libman Sacks Disease) OR ("Lupus Nephritis"[Mesh] OR Lupus Glomerulonephritis OR Nephritis, Lupus OR Lupus Nephritides OR Nephritides, Lupus OR Glomerulonephritis, Lupus OR Glomerulonephritides, Lupus OR Lupus Glomerulonephritides)) OR ("Lupus Vasculitis, Central Nervous System"[Mesh] OR Central Nervous System Lupus Vasculitis OR Systemic Lupus Erythematosis, Central Nervous System OR Central Nervous System Lupus OR central nervous system systemic lupus erythematosus OR Neuropsychiatric Systemic Lupus Erythematosus OR Lupus Meningoencephalitis OR Lupus Meningoencephalitides OR Meningoencephalitides, Lupus OR Meningoencephalitis, Lupus)) OR (systemic lupus erythematosus)) OR (lupus erythematosus)) OR (systemic lupus)) OR (Lupus Erythematosus, Discoid[MeSH Terms])) OR (Lupus Erythematosus, Cutaneous[MeSH Terms])	**83.756**

Table S3. The search strategy in Embase

#	Terms	Quotes
**9**	#6 AND #7 AND #8 AND ([chinese]/lim OR [english]/lim) AND [humans]/lim	**1.645**
**8**	('randomized controlled trial'/exp OR 'controlled trial, randomized' OR 'randomised controlled trial' OR 'randomized controlled trials' OR 'randomized controlled trials as topic' OR 'trial, randomized controlled' AND [embase]/lim) OR ('randomization'/exp OR 'random allocation' OR 'randomisation' AND [embase]/lim) OR ('double blind procedure'/exp OR 'double-blind method' OR 'double blind clinical trial' OR 'double blind comparison' OR 'double blind studies' OR 'double blind study' OR 'double blind test2' OR 'double blind trial' AND [embase]/lim)	**687.328**
**7**	'therapy'/exp OR 'prevention'/exp OR 'intervention':ti,ab,kw OR 'treatment':ti,ab,kw OR 'prevention':ti,ab,kw	**13.421.869**
**6**	#1 OR #2 OR #3 OR #4 OR #5	**115.502**
**5**	('lupus erythematosus nephritis'/exp OR 'glomerulonephritis lupoid' OR 'lupoid nephritis' OR 'lupus erythematosus nephritis' OR 'lupus glomerulonephritis' OR 'lupus kidney' OR 'lupus nephritis' OR 'lupus nephropathy' OR 'nephritis lupus erythematosus' OR 'nephritis systemic lupus erythematosus' OR 'systemic lupus erythematosis, nephritis') AND [embase]/lim	**17.968**
**4**	('systemic lupus erythematosus'/exp OR 'dermatovisceritism malignant' OR 'disseminated lupus' OR 'disseminated lupus erythematodes' OR 'disseminated lupus erythematosis' OR 'disseminated lupus erythematosus' OR 'erythematodes visceralis' OR lupovisceritis OR 'lupus erythematodes disseminatus' OR 'lupus erythematosus disseminatus' OR 'lupus erythematosus visceralis' OR 'lupus erythematosus systemic' OR 'osler libman sacks disease' OR 's.l.e.' OR 'sle' OR 'systemic lupus erythematodes' OR 'systemic lupus erythematosis' OR 'systemic lupus erythematous') AND [embase]/lim	**99.836**

**3**	'brain vasculitis'/exp OR 'angiitis brain' OR 'arteritis brain' OR 'brain angiitis' OR 'brain arteritis' OR 'cerebral arteritis' OR 'cerebral vasculitis' OR 'lupus vasculitis central nervous system' OR 'vasculitis brain' OR 'vasculitis central nervous system' AND [embase]/lim	**3.364**
**2**	'lupus'/exp OR 'discoid lupus erythematosus' OR 'cutaneous lupus erythematosus' AND [embase]/lim	**7.793**
**1**	('systemic lupus erythematosus'/exp OR 'dermatovisceritism malignant' OR 'disseminated lupus' OR 'disseminated lupus erythematodes' OR 'disseminated lupus erythematosis' OR 'disseminated lupus erythematosus' OR 'erythematodes visceralis' OR lupovisceritis OR 'lupus erythematodes disseminatus' OR 'lupus erythematosus disseminatus' OR 'lupus erythematosus visceralis' OR 'lupus erythematosus systemic' OR 'osler libman sacks disease' OR 'sle' OR 'systemic lupus erythematodes' OR 'systemic lupus erythematosis' OR 'systemic lupus erythematous') AND [embase]/lim	**99.638**

Table S4. The search strategy in Cochrane Library

#	Terms	Quotes
**1**	(therapy):ti,ab,kw	**704.834**
**2**	MeSH descriptor Therapeutics explode all trees	**143**
**3**	(intervention):ti,ab,kw	**379.140**
**4**	(treatment):ti,ab,kw	**782.201**
**5**	(prevention):ti,ab,kw	**182.665**
**6**	MeSH descriptor Treatment Outcome explode all trees	**3.459**
**7**	(#1 OR #2 OR #3 OR #4 OR #5 OR #6)	**1.219.409**
**8**	MeSH descriptor Lupus Erythematosus, Systemic explode all trees	**48**
**9**	MeSH descriptor Lupus Nephritis explode all trees	**11**
**10**	MeSH descriptor Lupus Vasculitis, Central Nervous System explode all trees	**2**
**11**	MeSH descriptor Lupus Erythematosus, Cutaneou explode all trees	**0**
**12**	(Lupus Erythematosus, Systemic):ti,ab,kw	**2.267**
**13**	"Lupus":ti,ab,kw	**3.298**
**14**	(#8 OR #9 OR #10 OR #11 OR #12 OR #13)	**3.342**
**15**	(#7 AND #14)	**2.773**
**16**	pubmed:an OR embase:an	**1.078.710**
**17**	(#15 NOT #16)	**967**

Table S5. The search terms and specific filtering options used in the clinical trials registries

#	Terms	Quotes
**Using “lupus” as search criteria**		
**1**	**Filtering options set in advanced search function in ClinicalTrials.gov**<br>Study type: “Intervention”;<br>Current status: “Recruiting” OR “Active, not recruiting” OR “Completed” OR “Enrolling by invitation” OR “Not yet recruiting”	**480**
**2**	**Filtering options set in advanced search function in Chinese Clinical Trial Register**<br>Country: “China”;<br>Study type: “Intervention” or “Prevention” or “Treatment” or “Prognosis”;<br>Subjects recruitment: “Recruiting” OR “Completed” OR “Not recruiting”	**24**
**3**	**Filtering options set in advanced search function in International Standard Randomised Controlled Trial Number Register (ISRCTN)**<br>Trial status: “Completed” OR “On going”<br>Recruitment status: “Recruiting” OR “No longer recruiting”	**183**

**4**	**Filtering options set in advanced search function in Australian and New Zealand Clinical Trials Registry (ACTR)**<br>Study type: “Intervention”;<br>Registry: ANZCTR<br>Allocation to intervention: “Randomised”;<br>Current status: “Recruiting” OR “Active, not recruiting” OR “Completed” OR “Not yet recruiting”	**12**

Appendix 4 Literature search and selection

Figure S1. Literature search and selection from published articles

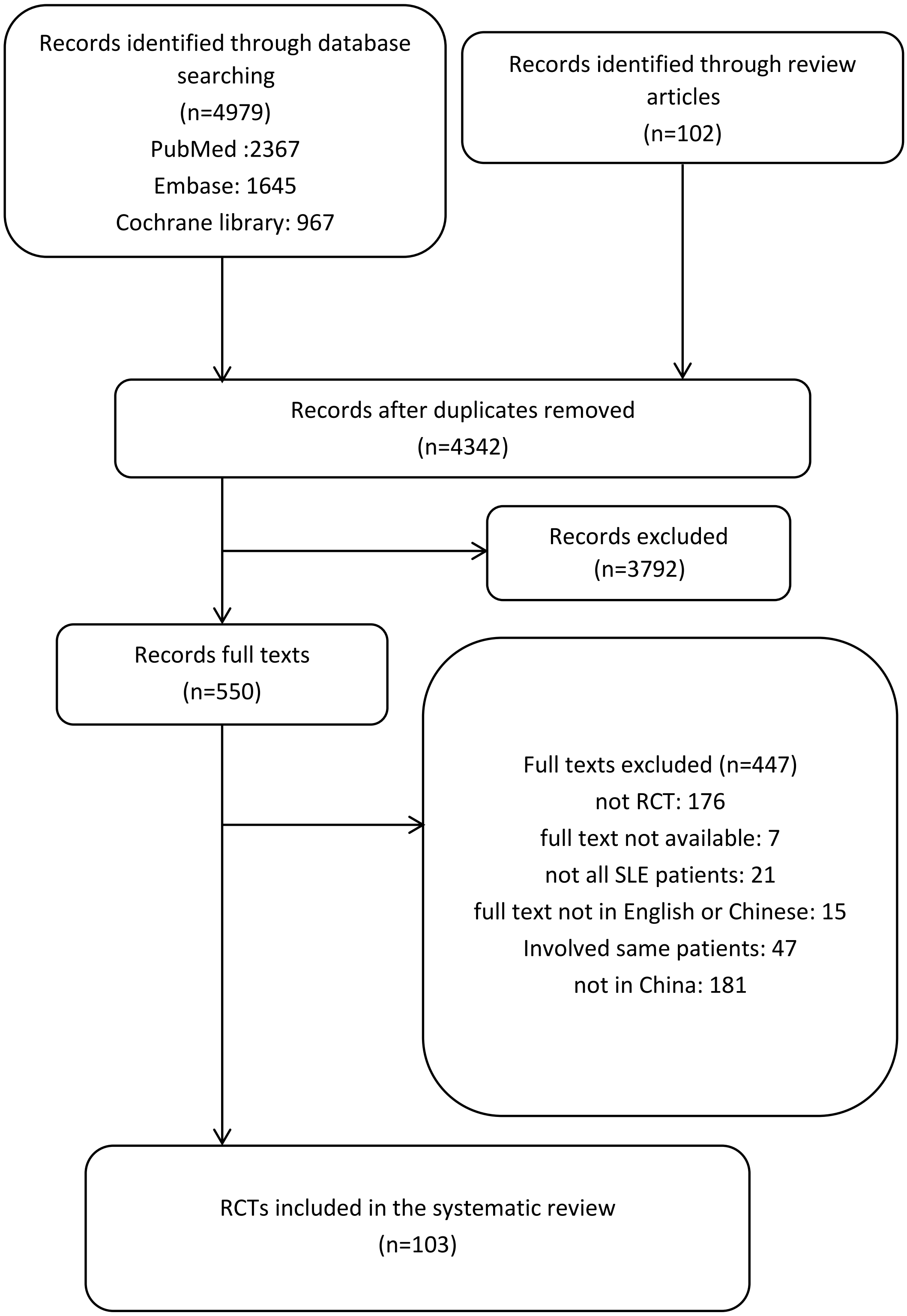

Figure S2. Record search and selection from clinical trials registries

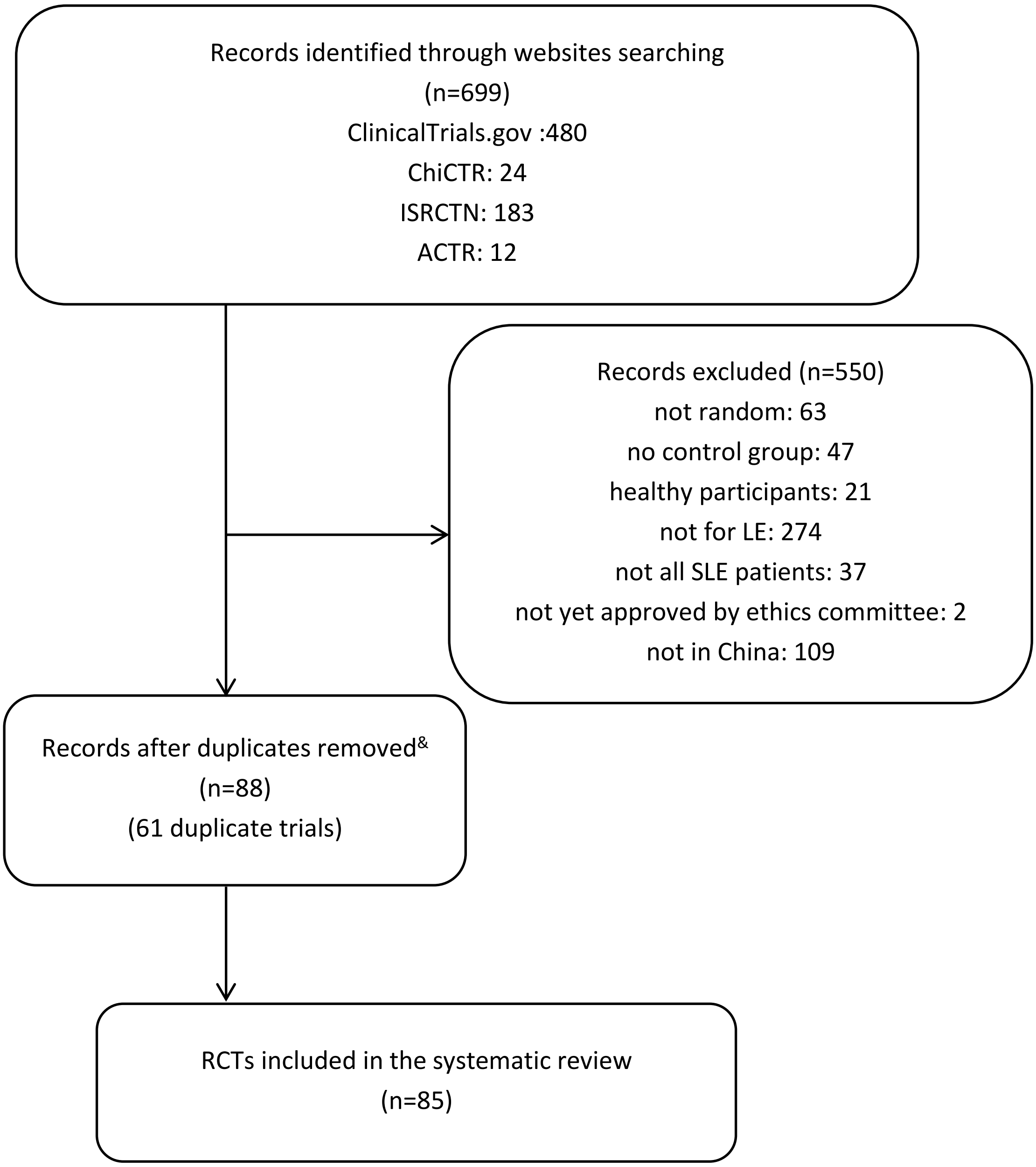

The searching term “lupus” was used in each clinical trial registries.

& Records from clinical trials registries and published articles were matched using registration ID or other information if registration ID was unavailable. 61 trials were duplicated in published articles with registry entries.

Appendix 5 Data abstraction form

Table S6. Data abstraction form

No.	Data Source	Author, year of publication	Registration ID	Year of start	Multinational study	Affiliation of primary investigator	Single or Multicenter	Subject categories	Number of participants

Continued table 6. Data abstraction form

Number of participants loss to follow-up	Percentage of loss-to-follow-up (%)	Intervention categories	Interventions	Intervention duration (month)	Age duration (years)	Blinding	Primary outcome	References	Country or area

Appendix 6. Characteristics of included RCTs

Table S7. Characteristics of included RCTs of LE conducted in China

**Categories**	**No (%)**
**Data source**	
Published articles	103 (54.8)
Clinical trials registries	85 (45.2)
**Center**	
Single center	96 (51.1)
Multiple centers	92 (48.9)
**Year of start**	
Before 2002	14 (7.4)
2002-2011	61 (32.4)
2012-2021	94 (50)
Not available	19 (10.1)
**No. of participants**	
<50	44 (23.4)
50-99	56 (29.8)
100-199	28 (14.9)
200-499	46 (24.5)
≥500	14 (7.4)
**Subjects**	
LN	57 (30.3)
**Blinding**	
Single blind	7 (3.7)
Double blind	51 (27.1)
Open label	44 (23.4)
Others	32 (17.0)
Others (Quadruple)	29 (15.4)
Others (Triple)	3 (1.6)
Not mentioned	54 (28.7)
**Intervention**	
Pharmacological treatment	170 (94.1)
Traditional Chinese medicine pharmacological treatment	35 (18.6)
Behavioral intervention	1 (0.5)
Biological therapy	9 (4.8)
Procedure	2 (1.1)
Others	3 (1.6)
Care	3 (1.6)
**Intervention duration (months)**	
<1	2 (1.1)
1-2.9	10 (5.3)
3-5.9	22 (11.7)

6-8.9	52 (27.7)
9-11.9	6 (3.2)
12-23.9	57 (30.3)
24-47.9	21 (11.2)
≥48	5 (2.7)
Not mentioned	13 (6.9)
**Primary outcome identification**	
Yes	143 (76.1)
No	45 (23.9)
**Trial registration** &	
Yes	127 (67.6)
No	61 (32.4)

Appendix 7 Risk of bias assessments

Figure S3. Risk of bias assessment graph: review authors' judgements (Low, Unclear and High) for each risk of bias item

Author and year	D1	D2	D3	D4	D5	Overall
Yang et al., 1996	!	-	!	-	+	-
Fu et al., 1998	+	-	-	-	+	-
Li et al., 2014	!	-	!	-	-	-
Wang et al., 2012	!	-	!	-	-	-
Wen et al.,2007	!	-	!	-	-	-
Yang et al., 2014	!	-	!	-	-	-
Chan et al., 2000	+	-	+	+	+	-
Liu et al., 2003	+	-	+	-	+	-
Liu et al.,2009	!	-	!	-	-	-
Wang et al., 2006	+	-	!	-	-	-
Guo et al., 2002	!	-	!	-	-	-
Wen et al.,2001	!	-	!	-	-	-
Wu et al., 2019	+	-	+	+	+	-
Qi et al.,2006	!	-	!	-	-	-
Cheng et al., 2005	+	-	!	-	-	-
Shi et al., 2007	!	-	!	-	-	-
Su et al.,2007	!	-	!	-	-	-
Tam et al., 2004	+	+	+	+	+	+
Tao et al.,2007	!	-	!	-	-	-
Liu et al., 2018	!	-	+	-	-	-
Liu et al.,2008	!	-	!	-	-	-
Qian et al., 2015	!	-	+	-	-	-
Li et al., 2002	!	-	!	-	-	-
Wang et al., 2007	+	-	-	-	+	-
Zhang et al., 2014	+	-	+	-	+	-
Appel et al., 2009	+	+	+	+	+	+
Bao et al., 2008	!	-	+	-	+	-
Dooley et al.,2011	+	+	+	+	+	+
Mok et al., 2019	+	+	+	+	+	+
Mok et al., 2020	+	-	+	-	+	-
Wu et al.,2003	!	-	!	-	-	-
Yap et al., 2012	!	-	-	-	+	-
Zhou et al., 2017	!	-	!	-	-	-
Chen et al., 2011	!	-	-	-	+	-
Liu et al.,2011	!	-	!	-	-	-
Chang et al., 2016	+	-	+	-	+	-
Furie et al., 2014	+	+	+	+	+	+
Ginzler et al., 2012	+	+	+	+	+	+
Li et al., 2009	+	-	-	-	+	-
Liao et al., 2011	+	+	+	+	+	+
Ma et al.,2014	!	-	!	-	-	-
Meng et al.,2014	!	-	+	-	-	-
Navarra et al., 2011	+	+	+	+	+	+
NCT00423098	!	-	+	-	+	-
Wang et al., 2015	!	-	-	-	+	-
You et al., 2009	+	+	+	+	-	-
You et al., 2010	+	+	+	+	+	+
Isenberg et al., 2015	+	+	+	+	+	+
Li et al., 2012	!	-	-	-	+	-
NCT00705367	+	+	+	+	+	+
Song et al.,2013	!	-	!	-	+	-
An et al., 2019	+	-	-	-	+	-
Boedigheimer et al., 2017	+	+	+	+	+	+

+ Low risk
! Some concerns
- High risk

D1 Randomisation process
D2 Deviations from the intended interventions
D3 Missing outcome data
D4 Measurement of the outcome
D5 Selection of the reported result

Huang et al., 2013	+	+	+	+	-	-
Liu et al., 2006	+	-	!	-	+	-
Clowse et al., 2017	+	+	+	+	+	+
Furie et al., 2015	+	+	+	+	+	+
Li et al., 2010	+	+	+	+	+	+
Zhang et al., 2015	+	-	+	-	-	-
Zhang et al., 2017	+	-	+	-	+	-
Furie et al., 2017	+	+	+	+	+	+
Merrill et al., 2016	+	+	+	+	+	+
NCT01408576	!	-	-	-	!	-
Stohl et al., 2017	+	+	+	+	+	+
Wallace et al., 2016	+	+	+	+	+	+
Zhang et al., 2018	+	+	+	+	+	+
Cheng et al., 2018	+	+	+	+	+	+
Deng et al., 2017	+	+	+	+	+	+
Feng et al., 2014	!	-	-	-	+	-
Furie et al., 2020	+	+	+	+	+	+
NCT01689350	!	-	+	-	+	-
Sheikh et al.,2021	+	+	+	+	+	+
Wang et al.,2011	!	-	+	-	-	-
Chatham et al., 2020	+	-	-	-	!	-
Geng et al., 2016	!	-	!	-	-	-
Kahl et al., 2016	+	+	+	+	+	+
Merrill et al., 2018	+	+	+	+	+	+
Zhang et al., 2019	+	-	!	-	+	-
NCT02141672	+	+	+	+	+	+
NCT02265744	+	+	+	+	+	+
Furie et al., 2019	+	+	+	+	+	+
He et al., 2019	+	+	!	+	+	!
Jordan et al., 2020	+	+	+	+	+	+
NCT02349061	+	+	+	+	+	+
NCT02437890	+	+	+	+	+	+
Wu et al.,2009	!	-	!	-	-	-
Zhou et al., 2017	!	-	!	-	-	-
Houssiau et al., 2020	+	+	+	+	+	+
Liu et al., 2015	+	-	-	-	+	-
NCT02708095	+	+	+	+	+	+
Sun et al., 2020	+	+	+	+	+	+
Xie et al., 2018	+	-	+	+	-	-
NCT02908100	+	+	+	+	+	+
NCT03021499	+	+	+	+	+	+
Zhang et al., 2019	+	-	!	-	+	-
NCT04136145	!	-	+	-	+	-
Cai et al., 2006	!	-	!	-	-	-
Chan et al., 2005	+	-	+	-	+	-
Chang et al., 2002	+	+	+	+	+	+
Chang et al., 2004	+	+	+	+	+	+
Chu et al., 2019	!	-	!	-	-	-
Kung et al., 1999	+	-	+	-	+	-
Li et al., 2005	!	-	-	-	-	-
Mok et al., 2005	!	-	+	-	+	-
Mok et al., 2011	+	+	+	+	+	+
Tang et al., 1997	+	+	+	+	+	+
Wu et al.,1998	!	-	!	-	-	-
Yang et al., 2003	!	-	!	-	-	-
Yang et al., 2005	!	-	!	-	-	-
Yin et al., 1994	!	-	!	-	-	-
Zeher et al., 2011	+	-	!	-	+	-
Zhang et al., 2011	+	-	+	-	+	-
Zhang et al., 2020	+	-	+	-	-	-
Zhao et al., 2016	+	-	+	+	+	-
Zheng et al., 2005	!	-	!	-	-	-
